# Value of positron emission tomography in diagnosing synchronous penile metastasis from urothelial bladder cancer

**DOI:** 10.1186/s12957-015-0696-1

**Published:** 2015-09-17

**Authors:** M. Rouanne, A. Alhammadi, D. Vilain, C. Radulescu, T. Lebret

**Affiliations:** Department of Urology, Hôpital Foch, 40, rue Worth, 92150 Suresnes, France; UFR des Sciences de la Santé, Versailles-Saint-Quentin-en-Yvelines University, Versailles, France; Department of Nuclear Medicine, Hôpital Foch, Suresnes, France; Department of Pathology, Hôpital Foch, Suresnes, France

**Keywords:** ^18^F-FDG PET/CT, Penile metastasis, Urothelial carcinoma, Bladder cancer staging

## Abstract

Metastases to the penis are extremely rare events. Most frequently, penile metastases come from the urogenital system (bladder, prostate) or the rectum-sigmoid colon. Usually painful, penile lesions may be asymptomatic, making diagnosis more challenging. Hence, we report the adding value of ^18^F-fludeoxyglucose–positron emission tomography/computed tomography (^18^F-FDG PET/CT) in the detection of penile metastases originating from urothelial carcinoma of the bladder. Arguably, penile metastases must be considered as an advanced disease requiring essentially palliative care. Therefore, accurate staging of clinically localized muscle-invasive bladder cancer is crucial to avoid useless curative intent radical surgery.

## Background

As bladder cancer management depends essentially on the extent of the disease, accurate clinical staging is crucial to select the most effective treatment [1]. Although penile lesions are mostly revealed by a variety of symptoms, some patients may be asymptomatic making diagnosis more challenging. Hence, we report the adding value of ^18^F-fludeoxyglucose–positron emission tomography/computed tomography (^18^F-FDG PET/CT) as a valuable method in the detection of penile metastases originating from urothelial carcinoma of the bladder.

## Case presentation

**Fig. 1:**
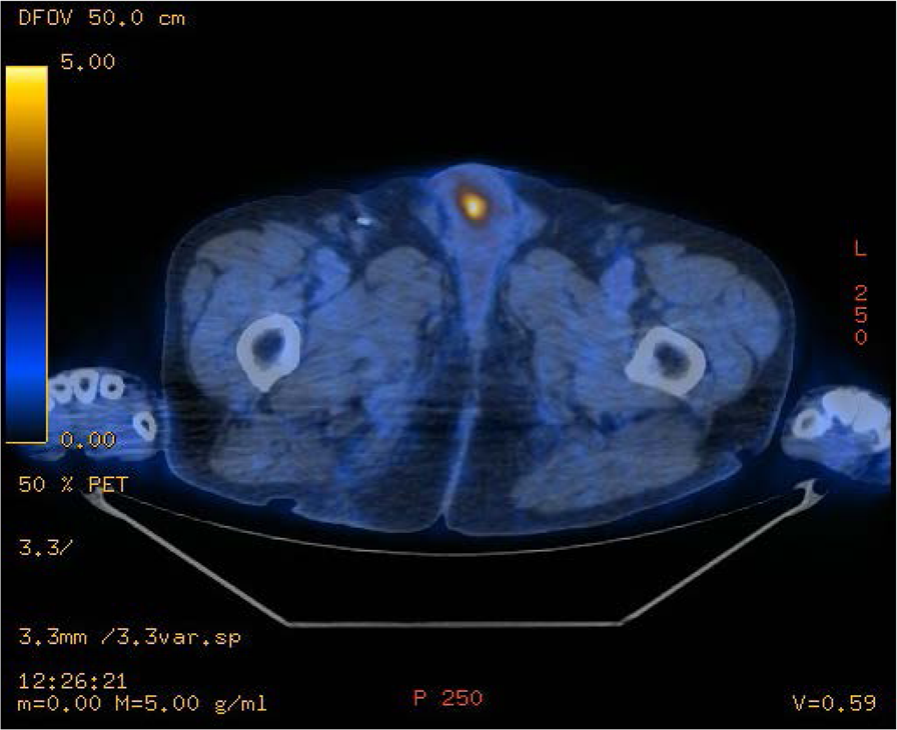
PET image showing high ^18^F-fluorodeoxyglucose uptake in the right corpus cavernosum

**Fig. 2:**
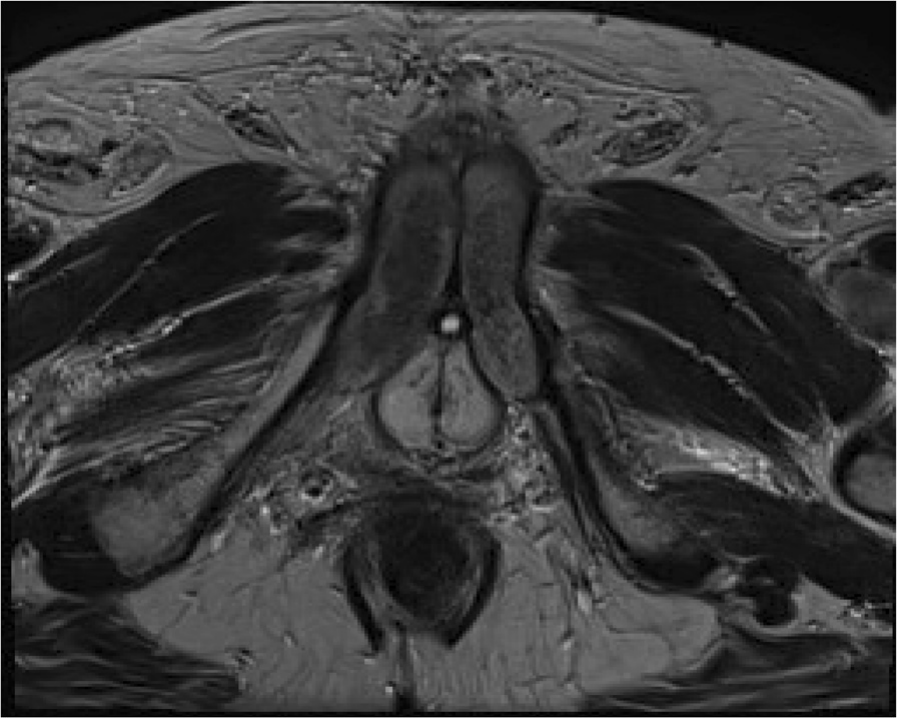
Hyposignal intensity in the right corpus cavernosum on a perineal axial T2-weighted slice

**Fig. 3:**
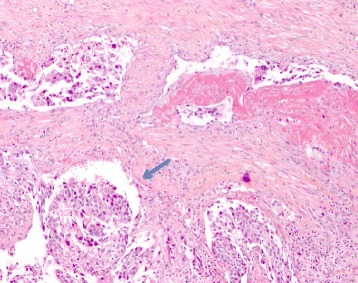
Carcinomatous tumoral emboli from urothelial carcinoma of the bladder occupying the erectile vascular tissue in the corpus cavernosum

A 78-year-old man presented with macroscopic hematuria, urinary frequency, and accidental leakage of urine. His past medical history only included benign prostatic hyperplasia. Initial assessment by fibroscopy showed a unique tumor developed on the anterior wall of the bladder. The patient underwent transurethral resection of the bladder tumor. Pathological analysis reported high-grade muscle-invasive urothelial carcinoma of the bladder (pT2 stage). The uro-computed tomography (CT) scan did not show any abnormality regarding the upper urinary tract. Metastatic workup with thoracic and abdomino-pelvic CT scan showed clinically localized bladder cancer. The patient was then referred to our department to undergo a curative intent surgery according to the multidisciplinary committee recommendations. Bilateral extended pelvic lymphadenectomy with radical cystoprostatectomy and ileal neobladder reconstruction were performed. Definitive pathological analysis rendered pT2N0M0 high-grade urothelial carcinoma (TNM 2009) with free surgical borders, associated with prostatic adenocarcinoma Gleason score 7 (3 + 4), grade 4 involving 20 % of both prostatic lobes. A total of 15 days after surgery, the patient developed penile pain. Physical examination showed painful induration located on the right corpus cavernosum of the penis. PET/CT imaging showed a high focal intense uptake of ^18^F-FDG in the right corpus cavernosum of the penis (Fig. 1). The report concluded that there was a high suspicion of metastatic spread to the penis. Complementary penile magnetic resonance imaging (MRI) associated with simultaneous fine needle biopsy were realized. Penile MRI showed low to intermediate signal intensity as compared with the surrounding corpus cavernosum on an axial T2-weighted image (Fig. 2). Histopathological examination from the right corpus cavernosum biopsy revealed carcinomatous tumor emboli from the urothelial carcinoma of the bladder (Fig. 3). The patient was referred to the medical oncologist for appropriate treatment of the metastatic disease. Concomitant radio-chemotherapy was decided according to the genitourinary tumor board recommendations. This treatment included local radiation associated with cisplatin-based regimen chemotherapy. The patient received chemotherapy with a combination of gemcitabine and carboplatin followed by radiotherapy. Despite such intensive combined treatment, metastatic cancer progression occurred and the patient died.

### Discussion

Penile metastases are extremely rare events. The most common primary tumors concern the urogenital system (i.e., bladder, prostate, and kidney) or the rectum-sigmoid colon [2–4]. Indeed, physiopathology of metastases to the penis is still unclear. Several mechanisms of dissemination have been described. A usual way of spread seems to be the retrograde venous dissemination from the pudendal venous system into the dorsal venous system of the penis. However, alternative ways have been evocated such as retrograde lymphatic spread into penile lymphatic channels, iatrogenic implantation secondary to instrumentation, or direct extension from the primary tumor [2–4]. Interestingly, most cases of penile metastasis are metachronous. The time interval between primary tumor and penile metastasis ranges from 3 to 60 months. Indeed, about two thirds of all penile metastases are diagnosed at a meantime of 18 months after the detection of the primary tumor [5]. Moreover, penile metastases are usually symptomatic. Most frequent are malignant priapism due to the tumor infiltration of the corpus cavernosum (>40 %), intense penile or perineal pain (≈10 %), penile nodules or ulceration, generalized swelling of the penis, and various urinary symptoms [2–4]. As previously reported, penile metastases from urothelial carcinoma of the bladder may be detected by either pelvic CT scan or MRI [2–8]. Classically, the corpus cavernosum is the site of involvement of metastatic penile carcinoma whereas the glans penis and corpus spongiosum are rarely involved [6]. Even though MRI may able to distinguish metastatic lesion from primary tumor of the penis, the presence of penile lesion must be pathologically confirmed. Percutaneous biopsies are also possible using penile block as anesthesia [7]. However, as presented in our case report, penile metastasis may be asymptomatic without any clinical sign of the lesion at the time of staging. Indeed, abdomino-pelvic MRI and/or CT scan do not systematically include the penis in routine clinical practice. In this context, diagnosis may be more tricky and challenging. FDG/PET-CT analysis may represent a valuable non-invasive technique which correctly identifies such lesions providing an extensive staging in a single session. Although ^18^F-FDG/PET-CT has been shown to improve baseline staging in the nodal staging of bladder cancer [1], controversy stills exists in relation to the widespread use of PET/CT in clinical practice [9]. One of the main reasons is its additional cost for a test with low sensitivity [1].

Definitely, accurate staging of bladder cancer is crucial to guide physicians in establishing the best therapeutic strategy and to avoid useless curative intent treatment option. Once penile metastasis has been diagnosed, the prognosis is poor and death occurs rapidly [2]. The average survival in patients presenting with penile metastasis is very short (i.e., 4 months from diagnosis), as it is highly associated with advanced disease [3]. Conversely, only a few cases of synchronous metastasis have been described. Overall, penile metastases must be considered as a non-curable advanced multi-systemic disease requiring palliative therapy such as local radiotherapy and systemic chemotherapy. Partial or total penectomy may be discussed in order to palliate painful symptoms. However, curative intent surgery should be avoided. In our case report, metastasis became evident 15 days after surgery, which is very prompt. Indeed, the patient was asymptomatic and the abdomino-pelvic CT scan did not show any abnormality at baseline staging. Hence, we hypothesize that penile metastasis was already present at time of surgery. Arguably, this case report strengthens the impact of ^18^F-FDG/PET-CT for detecting asymptomatic penile metastasis originating from muscle-invasive urothelial carcinoma of the bladder.

## Conclusions

Despite its rarity, diagnosis of metastatic lesion to the penis may be challenging particularly in asymptomatic patients. Synchronous penile metastases should be considered as a multi-systemic disease with poor prognostic. Thus, unncessary curative intent radical cystectomy might be avoided. This report highlights the clinical value of ^18^F-FDG/PET-CT to detect penile metastases originating from urothelial carcinoma. Therefore, ^18^F-FDG-PET/CT may help physicians to define the best treatment strategy in patients with advanced bladder cancer.

## Consent

Written informed consent was obtained from the patient’s wife for publication of this case report and any accompanying images. A copy of the written consent is available for review by the Editor-in-Chief of the World Journal of Surgical Oncology.

## Abbreviations

^18^F-FDG PET/CT: ^18^F-fludeoxyglucose–positron emission tomography/computed tomography
CT: computed tomography
MIBC: muscle-invasive bladder cancer
